# Model transfer from 2D to 3D study for boxing pose estimation

**DOI:** 10.3389/fnbot.2023.1148545

**Published:** 2023-03-20

**Authors:** Jianchu Lin, Xiaolong Xie, Wangping Wu, Shengpeng Xu, Chunyan Liu, Toshboev Hudoyberdi, Xiaobing Chen

**Affiliations:** Faculty of Computer and Software Engineering, Huaiyin Institute of Technology, Huai’an, China

**Keywords:** boxing robot, computer vision, human pose estimation, 3D model transfer, negative transfer

## Abstract

**Introduction:**

Boxing as a sport is growing on Chinese campuses, resulting in a coaching shortage. The human pose estimation technology can be employed to estimate boxing poses and teach interns to relieve the shortage. Currently, 3D cameras can provide more depth information than 2D cameras. It can potentially improve the estimation. However, the input channels are inconsistent between 2D and 3D images, and there is a lack of detailed analysis about the key point location, which indicates the network design for improving the human pose estimation technology.

**Method:**

Therefore, a model transfer with channel patching was implemented to solve the problems of channel inconsistency. The differences between the key points were analyzed. Three popular and highly structured 2D models of OpenPose (OP), stacked Hourglass (HG), and High Resolution (HR) networks were employed. Ways of reusing RGB channels were investigated to fill up the depth channel. Then, their performances were investigated to find out the limitations of each network structure.

**Results and discussion:**

The results show that model transfer learning by the mean way of RGB channels patching the lacking channel can improve the average accuracies of pose key points from 1 to 20% than without transfer. 3D accuracies are 0.3 to 0.5% higher than 2D baselines. The stacked structure of the network shows better on hip and knee points than the parallel structure, although the parallel design shows much better on the residue points. As a result, the model transfer can practically fulfill boxing pose estimation from 2D to 3D.

## 1. Introduction

Boxing as a strenuous exercise is gradually being accepted by the general public in China. It has been promoted in many universities and has relevant professional courses (Xu, 2018; Li L., 2019; Li X., 2019; Logan et al., 2019). It can improve citizens’ and students’ physical and mental health (Tjønndal, 2019), and even enhances the self-protection abilities of women (Hu, 2018; Fuerniss and Jacobs, 2020). However, this results in a new problem of a coach shortage. Many researchers have tried to employ computer vision and robot technology to solve the shortage problem of coaches (Huang et al., 2019; Li et al., 2021; Lin et al., 2021, 2022; Mendez et al., 2022). The human pose estimation technology can predict boxing elements for better teaching, which can reduce reliance on coaches and increase entertainment in boxing training.

Currently, the 3D camera can provide more depth information than the traditional 2D RGB camera. This advantage can help in the advancement of many tasks of image processing, such as MRI images (Chen et al., 2019; Wu G. et al., 2022), robots (Song et al., 2020), 3D faces (Ning et al., 2020; Wu H. et al., 2022), and so on. 3D human pose estimation becomes a cutting-edge and interesting direction. Many researchers have attempted to reconstruct a 3D human pose estimation with 2D or 3D cameras. Since 2D estimation has been researched comprehensively and wholistically (Wang et al., 2021), it will be crucial to determine whether 2D estimation is compatible with 3D estimation. Adapting the existing 2D models to the application with 3D cameras and studying the advantages of these models is essential to boxing applications and promoting the technology of human pose estimation.

In the field of artificial intelligence, there are two main ways for human pose estimation: bottom-up and top-down (Xiao et al., 2018; Wang et al., 2021). For instance, the top-down method detects each person first, and then directly detects the key points of each person. It is a two-stage method. Most research is based on 2D imagery and shows brightness design and theoretical structures that achieve SOTA results, such as Hourglass (HG) models (Newell et al., 2016; Xiao et al., 2018; Hua et al., 2020; Xu and Takano, 2021), and High Resolution (HR) networks (Sun et al., 2019; Yu et al., 2021; Xu et al., 2022). In contrast, the bottom-up method recognizes the limbs of people at the beginning and groups these limbs for each person, such as in the OpenPose(OP) models (Cao et al., 2017) and Hourglass(HG) models (Nie et al., 2018). Three mainstream models of the OP, HG, and HR networks are suitable for our boxing application. However, the problem of channel inconsistency directly affects the transfer of a 2D model to a 3D image. The basic popular methods need to be investigated deeply, and it is important to reveal their performance differences in detail for better improvement.

Model transfer technology is employed to help improve the application of human pose estimation by transferring their models and parameters. The performance of estimation of boxing poses is evaluated on RGBD image. The main contributions of this paper are:

• The depth channel is patched by different strategies when data input is inconsistent, which illustrates that the negative transfer can happen in this step, and it implies that the machine learning method can further improve the strategy.
• A detailed analysis of human pose estimation technology reveals the advantages and disadvantages of mainstream models used in boxing pose estimation, indicating the new improving direction of this technology.
• The model transfer from 2D to 3D images is studied for boxing practice, which shows that 2D models can be compatible with the 3D inputs of 3D cameras.

In this manner, the three mainstream models of the OP, HG, and HR networks are studied. The following sections are mainly divided into three parts: (1) Related work. Research work about human pose estimation is presented and analyzed. The important structures of neural networks are discussed; (2) Method. The model-transfer technology is employed to study the transfer of relative top-down and bottom-up models, respectively. 2D inputs are transferred to adaptive 3D inputs. This section also describes different ways for model transfer. (3) Results and discussion. The previously mentioned approaches are carried out after model transfer, and 3D and 2D transfer results are analyzed in detail. Three basic methods are discussed to analyze their existing problems.

## 2. Related work

### 2.1. Top-down way

Newell et al. (2016) proposed the HG method, which expanded the ResNet structure to realize the extraction of pose information. To improve the joint position regression, Xiao et al. (2018) added a few deconvolutional layers over the last convolution stage in the ResNet, which generated heatmaps from deep and low-resolution features. Considering Xiao’s architecture, Moon et al. (2019) designed a PoseFix network to refine the estimation, which applies to a model-agnostic pose refinement method. Hua et al. (2020) took a multipath affinage way to improve HG networks. Furthermore, Graph stacked HG network was developed by Xu and Takano (2021). It has an HG shape consisting of a chain of convolution and up-convolution layers followed by a regression part for generating a 3D pose. However, this estimation is based on 2D image inputs.

Chen et al. (2018) proposed a cascaded pyramid network (CPN) for human pose estimation. It has GloableNet and RefineNet as two parts, and each layer was parallel to exchange information. But for a better exchange of information between different scale features, Sun et al. (2019) further proposed a high-resolution (HR) network method for information exchange at the base of a huge pyramid structure. HigherHRNet was proposed (Cheng et al., 2020) to use the high-resolution feature pyramid for prediction by a 1 × 1 convolution to heatmaps based on the HR network. It can solve the scale variation challenge in bottom-up multi-person pose estimation. To reduce the parameters and improve speed, Yu et al. (2021) refined the HR network called the Lite-HR network, which applies shuffle blocks to the HR network. The accuracy got a slight drop. Xu et al. (2022) applied the transformer model to human pose estimation. This simple structure can be the backbone to extract features for the HR network. This improvement is based on longer training, and it has challenges for patch embedding when there is less information around key points.

### 2.2. Bottom-up way

The OP network model (Cao et al., 2017) based on the affinity field could simultaneously locate multiple people and get wild applications (Nakai et al., 2018; Viswakumar et al., 2019; Chen et al., 2020; Nakano et al., 2020). The key points are detected in each joint class and grouped into limbs between each joint point. With a part association field (PAF) restriction, the limbs are gathered at a minimum cost. Nie et al. (2018) proposed a pose partition network (PPN) to detect joints and regression for multi-persons, which is based on the HG network. Since a PAF was used to associate body parts with each other, a part intensity field (PIF) was proposed to localize body parts (Kreiss et al., 2019) and help form full human poses. Osokin (2018) proposed a lightweight OP network, which only remained in one refinement stage and replaced the VGG network with the MobileNet in the backbone. Wu et al. (2021) proposed a rapid OP network for astronaut operation attitude detection. They changed the original two-branch structure to a single-branch structure, which improved the calculation speed. Geng et al. (2021) proposed disentangled key point regression (DEKR), which uses a multi-branch structure for separate regressions to get the key points in the bottom-up paradigm. For multi-person pose estimation, Jin et al. (2020) reformulated the task of multi-person pose estimation as a graph-clustering problem. The OP networks rely on the backbone network for feature extraction.

According to the above analysis, studies based on the OP, HG, and HR networks are very extensive, and the HR and HG networks can be used both in the top-down and bottom-up way. The study of these three methods can be better at comprehensively finding problems in our boxing sport application.

## 3. Materials and methods

### 3.1. Device and dataset

The image data collecting tool was a 3D Intel Realsense D455 camera with a 640 × 480 resolution. It was placed approximately 185 cm above the ground, with a depression angle of 10 degrees around. RGBD data was collected in various indoor environments such as classrooms and research labs. Five basic boxing poses, including punch, swing, hook, backward, and side slide were recorded in left and right ways. More than 100 students contributed to the collection. After data cleaning, 280 images were selected to form a dataset for this research. The dataset was randomly divided into three parts: a training set (120 images), a validation set (40 images), and a test set (120 images). These three sets didn’t have the same person.

### 3.2. Model transfer learning

After testing, three SOTA models of the OP, HG, and HR networks were studied. They were transferred as source models since these three basic models have been researched extensively and achieve each best performance. All the models are 2D inputs. The boxing pose estimation was the target learning task. The 640 × 480 boxing image is estimated directly because three models are studied under the same size of the input, feature maps, and heatmaps.

#### 3.2.1. OP network model

**FIGURE 1:**
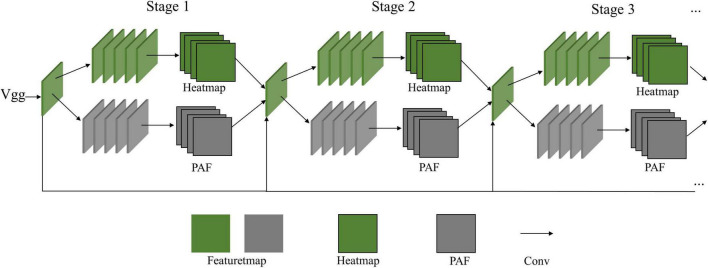
OP network model with a parallel steam structure of heatmaps and PAFs.

There are two primary parallel branches in the OP network. One branch is trained to predict the heatmap of human pose key points, and the other branch is trained to predict the PAF that can help organize the components of body limbs in a bottom-up way. The model repeats the basic branches several times as stages as displayed in Figure 1. This design can be easy for multi-person estimation as it estimates every person’s key point at one computation, but the heatmap branch is simple for extracting complex features and structures since it only depends on convolution layers.

#### 3.2.2. Stacked HG network model

**FIGURE 2:**
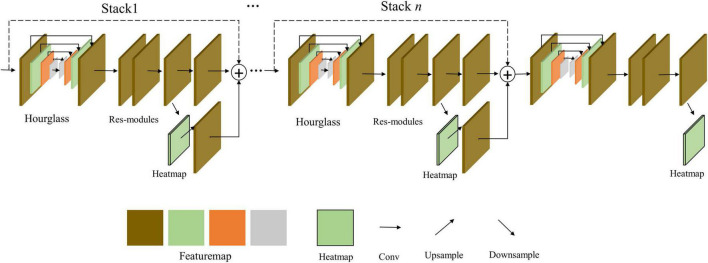
Stacked HG network with local and global contexts.

In Figure 2, the HG network is inspired by the pyramid structure to deal with the local and global context. In each stack, there is a pyramid structure integrated inside, and the heatmaps are generated to predict key points, and each stack is repeated to group a complex network. It can be seen that the learning ability is improved by its pyramid structure. The stack is very similar to the OP stage, so it can be used in both top-down and bottom-up ways, but there is less information exchange for each stack. This may cause limited learning in the local context.

#### 3.2.3. HR network model

**FIGURE 3:**
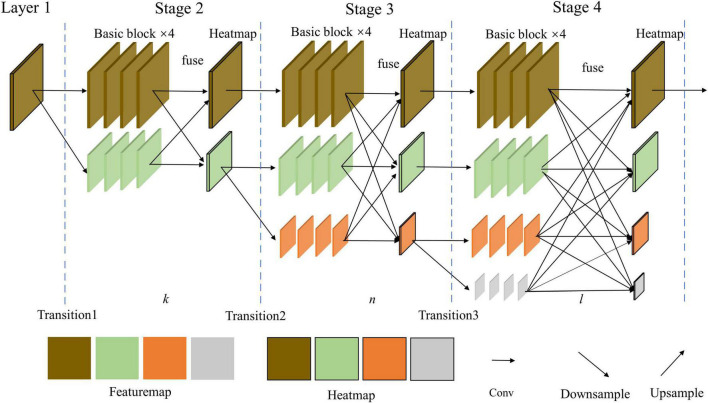
HR model with an inverse pyramid parallel structure and information exchange fuse.

As shown in Figure 3, the HR network has an inverse pyramid parallel structure compared to the HG network. It can be seen that there are 4 parallel channels in different image scales, which can get local and global information. Besides, this separates 3 or 4 stages to make the model better exchange information between different scale feature maps. The parallel branches in each stage are usually repeated a few times to make a better extraction. Therefore, features can be combined in multiple ways. Compared with the above two networks, the HR network in each stage doesn’t keep the same size, which means it may have less learning ability for symmetry structures.

**FIGURE 4:**
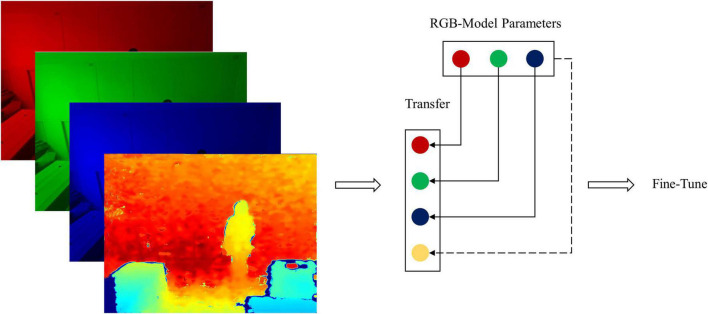
The RGB-D model transfer based on the RGB model.

The above three source models (*Model*_2D_) were transferred as shown in Figure 4. The input data in a source learning task can be described as$X_{S}=\left\{x_{1}^{n},x_{2}^{n},x_{3}^{n},\cdots ,x_{k}^{n}\right\}$, *n* is the input data dimension, and *k* is the instance number. In the target learning task, the input data is$X_{T}=\left\{x_{1}^{n+1},x_{2}^{n+1},x_{3}^{n+1},\cdots ,x_{k}^{n+1}\right\}$, and the instance number *m* is far less than *k*. Therefore, the posterior distribution of the source domain *P*_*S*_(*y*|*x^n^*) needs to change to the target domain posterior distribution *P*_*T*_(*y*|*x*^*n* + 1^). The fine-tuning method can be used to adapt the source posterior distribution to the target domain. To solve the problem of lacking depth channel in source models, an additional channel of the parameter was patched based on RGB channels as in formula (1):


(1) $$M\,{\text{odel}}_{3\,D}=M\,o\,d\,e\,l_{2\,D}\,\left(R,G,B\right)+P\,a\,r\,a\,m_{\text{channel}}\,$$

Where the channel parameters can be chosen from *R*, *G*, and *B* channels or the mean combination of these three channels. When transferring parameters from source models, the different channel effects should be examined, and the best way to improve the depth channel effects on predicting posture points should be determined. Boxing pose data were used to fine-tune the models to generate new models.

## 4. Results and discussion

The performance of transferred models was studied in four ways: (1) the average accuracy was obtained about boxing pose key point positions, and the corresponding accuracy of each model after the transfer of different channels was compared to baselines of 2D transfer; (2) the impact of fine-tuning instance amount on model transfer improvement; (3) the Flops and parameter number of each model were shown for evaluating model complex, and average cost times of models per image were compared; (4) a direct comparison of the pose estimation of boxing basic actions among different models, along with pose estimation display.

### 4.1. The average prediction accuracies of key points

There are seven distinct critical points for estimating human poses including the head, shoulder, elbow, wrist, hip, knee, and ankle. To keep the comparison of points consistent, the neck point of OP does not show here. Table 1 shows the average accuracy of 10 times repeat on each point recorded without fine-tuning, 2D transfer with fine-tuning, and 3D transfer with a different kind of channel. The source models of the OP, HR, and HG networks were pretrained and released publicly by their developers.

**TABLE 1:** The average accuracy of different key points.

Network	Key point	Head	Shoulder	Elbow	Wrist	Hip	Knee	Ankle	Total average
OP (%)	Without	82.3	89.9	80.2	72.6	71.7	91.4	92.8	83.0
	2D	**98.9**	**95.2**	**91.7**	**91.3**	**88.2**	**97.1**	**97.4**	**95.3**
	3D-R	98.6	96.2	89.7	88.9	85.7	95.8	97.4	93.2
	3D-G	97.9	94.7	85.2	86.3	86.7	95.3	97.6	92.0
	3D-B	97.3	96.4	86.8	90.6	85	97.2	97.4	93.0
	3D-Mean	**99.4**	**96.9**	**92.2**	**92.1**	**92.1**	**97.5**	**97.9**	**95.8**
HG (%)	Without	95.8	89.9	80.2	72.6	87.6	90.2	94.6	90.7
	2D	**99.7**	**95.2**	**91.7**	**91.3**	**92.9**	**99.2**	**97.5**	**96.1**
	3D-R	99.8	96.2	89.7	88.9	93.4	98.0	97.2	96.3
	3D-G	99.6	94.7	85.2	86.3	92.5	98.7	96.8	95.9
	3D-B	99.6	96.4	86.8	90.6	92.3	98.6	96.9	96
	3D-Mean	**99.8**	**96.9**	**92.2**	**92.1**	**93.8**	**99.5**	**98.9**	**96.5**
HR (%)	Without	94.5	98.7	91.6	81.2	79.5	97.5	98.7	91.7
	2D	**99.8**	**98.6**	**94.0**	**95.9**	**90.5**	**97.9**	**99.8**	**96.6**
	3D-R	99.5	97.5	85.0	91.7	85.4	93.1	96.1	92.6
	3D-G	99.6	97.9	91.4	93.6	86.5	97.2	97.4	96.9
	3D-B	96.2	98.3	85.0	93.5	86.4	93.5	96.7	92.8
	3D-Mean	**99.8**	**98.6**	**94.6**	**96.8**	**91.8**	**97.9**	**99.8**	**97.0**

Bold values represent the best results of 2D and 3D model transfer.

Table 1 shows that three models are listed in each column, and in each row, the average accuracy of each key point is compared. The 2D rows can be chosen as baselines for 3D transfer. In 3D transfers, the R, G, and B channels were examined, respectively. In addition, the mean of the three channels was also examined.

**FIGURE 5:**
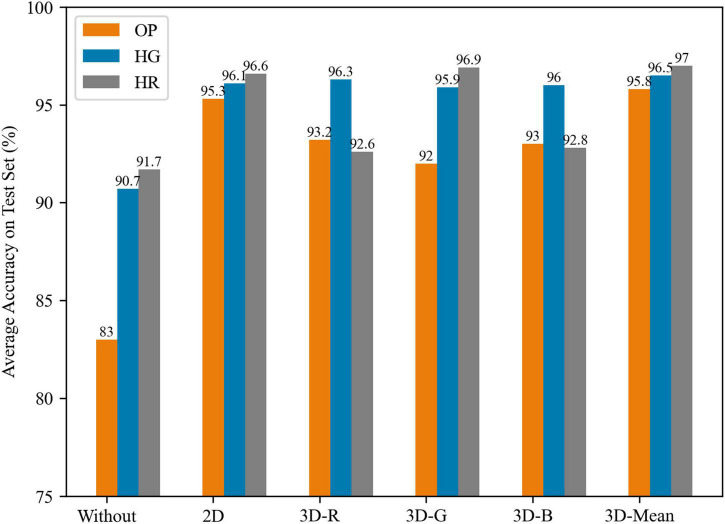
The average results of different transfer way by OP, HG, and HR models.

Each network in Table 1 includes 6 different methods of fine-tuning; 2D, and 3D are displayed in Figure 5. There are 6 groups for three networks. The accuracies of the first group are lower than those of the second group after finetuning in 2D transfer, and the HR network achieves the highest average accuracy of 96.6%. The HG network achieves a similar performance at 96.1%, while the OP network achieves 95.3%, which is increased by 12.3%. After fine-tuning, both networks perform slightly better than the OP network. When in 3D transfer, the third to sixth groups in Figure 5 show different channel parameters that are chosen or combined to patch the lacking depth channel. The R and B channels affect the OP and HG networks the most, whereas the G channel affects the HR network the most, and the R and B channels even can cause a negative transfer on the HR network (from 96.6 to 92.8%). The mean strategy of R, G, and B channels get the best estimation than in a single one-channel way. This situation shows that the three networks learn different patterns in different channels. The OP and HG network extracts features from three channels equally, while the HR network gets features from the G channel, which is highly related to depth information. Features from B and R channels are less related to depth information.

**FIGURE 6:**
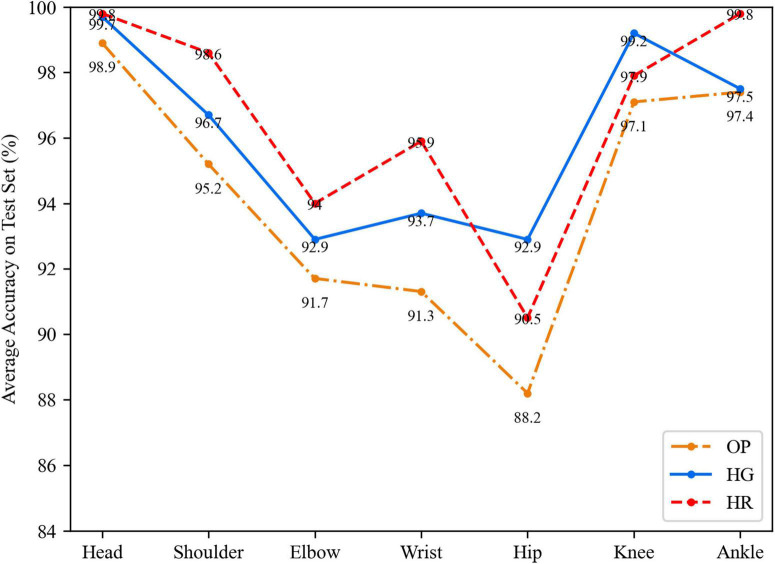
The average accuracy on each kind of key point in a 2D way.

Figure 6 shows the detailed results of 2D. The HR network can get the best estimation on the head, shoulder, elbow, wrist, and ankle points. However, the HG network estimates hip and knee points better than the HG network. Both the OP and HR networks are worse in hip point estimation. This phenomenon might be caused by the lower learning ability of the OP network and the lower symmetric ability of the HR network than the HG network since these two kinds of points have fewer texture features in the image. The HG model has a lower feature extraction than the HR network.

**FIGURE 7:**
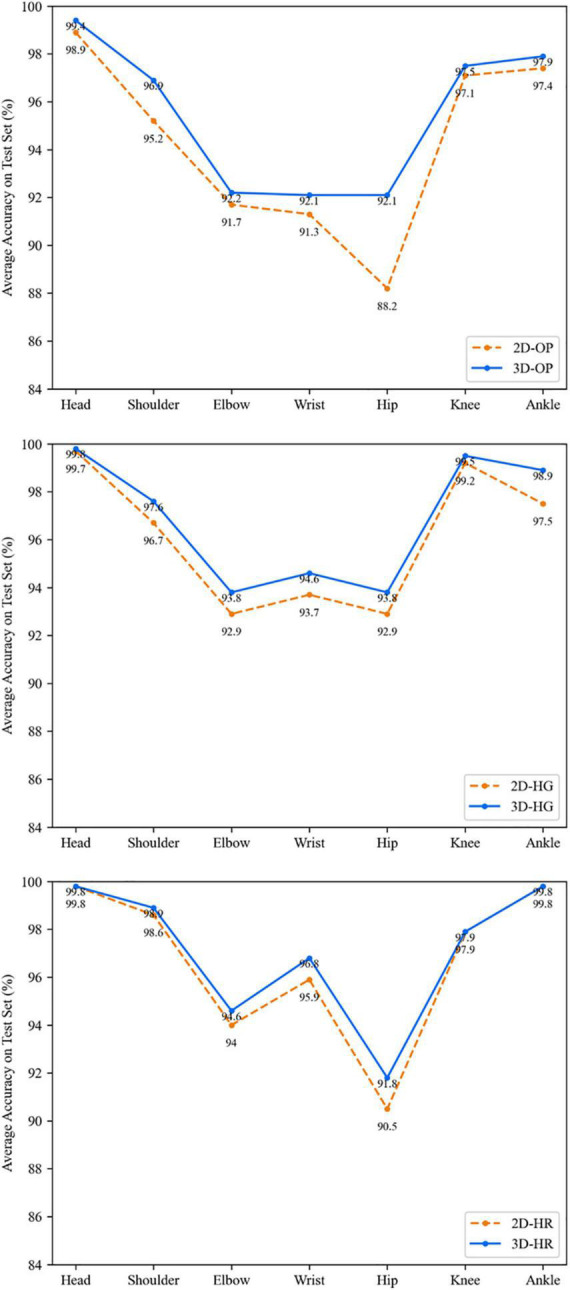
The detailed performance of different networks comparing 2D and mean of 3D.

**FIGURE 8:**
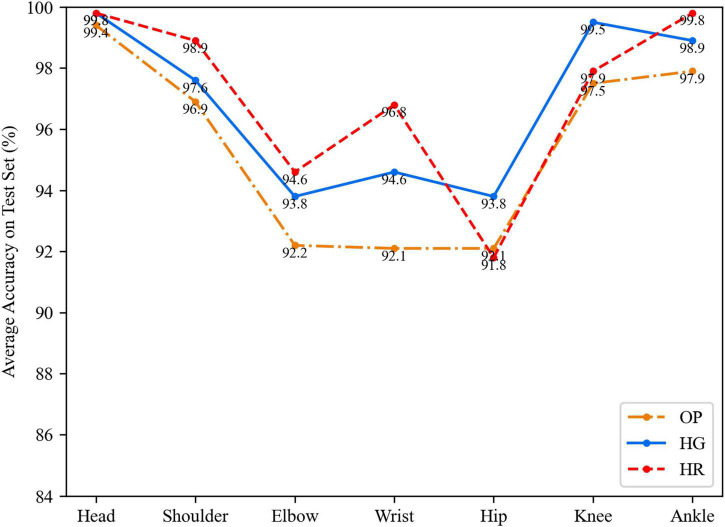
The average accuracy on each key point in the mean 3D way.

Finally, compared with the second group of 2D transfer, the mean 3D group average accuracies are all higher than that of the 2D group as shown in Figure 7. The OP network is improved by 3.3% on hip points, which is higher than other networks. This may be caused because of the previous imbalance training by authors. In Figure 8, the 3D transfer also shows a similar result as the 2D transfer. The HR network performs better on the head, shoulder, elbow, wrist, and ankle points, but the HG network performs better on hip and knee points. It means that the HR network has a deficiency on the hip and knee points when there is less texture information around. The depth channel shows less help to the estimation. This may be the bottleneck of transfer learning when lacking depth training data.

### 4.2. The fine-tuning effect of different training data set size

The average accuracy curves of the OP, HR, and HG networks are drawn under the different training dataset sizes from 40 to 120, which increases by 20 each step.

**FIGURE 9:**
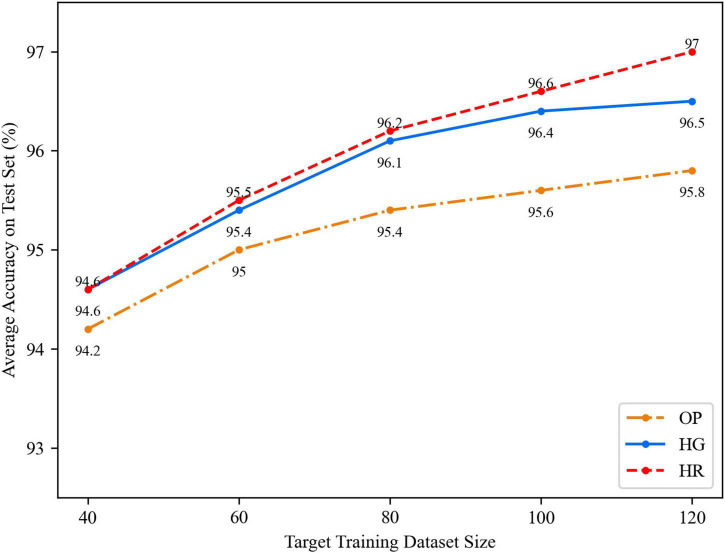
The total average accuracy of different networks after model transfer.

As shown in Figure 9, the horizontal axis indicates the amount of training dataset size. The vertical axis indicates the average accuracy on the test set. The results show the HR model still performs better than others. But when the data size is small, from 40 to 80, it is almost the same as the HG network. With an increase in the training dataset size, the HR network becomes better than the HG network. It means the HR network might get much better results when the dataset size becomes large. The OP network performance increases a bit slower than the other two networks and it tends to be plain.

### 4.3. The FLOPs and average cost time

The Floating-Point Operations (FLOPs) and average cost time on the test set are shown in Table 2. The same resolution images were fed into the three networks. The parameter number of each kind of network is displayed. It can be seen that the numbers of HG and OP’s parameters and average cost times are almost equal, and they are both nearly twice that of HR. As the parameters are reduced by half, the GFLOPs can be reduced largely.

**TABLE 2:** FLOPs and average time cost on each model.

Method	Input size	#Params	GFLOPs	Average cost time (s)
OP	640 × 480	52.3M	308.5	0.57
HG	640 × 480	53.1M	359.8	0.68
HR	640 × 480	28.5 M	48.08	0.34

### 4.4. Comparison of the pose estimation on boxing basic movements

**FIGURE 10:**
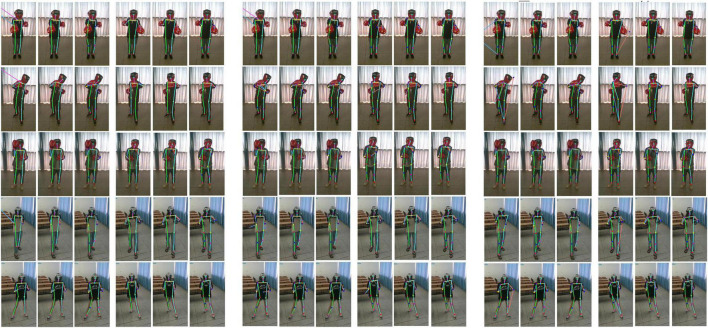
The pose estimation of the OP network **(left)**, HG network **(middle)**, and HR network **(right)** after model transfer.

Five postures of punch, swing, hook, backward, and side sliding are estimated in both left and right ways as displayed in Figure 10. The figure shows two different scenes, and the results are listed in a sequence of without fine-tuning, after the 3D transfer, and ground truth. It also displays the results of the OP, HG, and HR networks, respectively.

In the first column of each figure, there are many errors in five poses. The head and wrist points are wrongly located. The estimation results are littered randomly since the left and right results of both the OP and HG networks have a big difference. This phenomenon may be caused by the background. The edge of curtains or chairs might be like the human edge. The HR network’s left and right results are more symmetric than other methods. Besides, the obscuring from the boxing helmet and the camera’s inconsistent view also result in locating the wrong place such as the ankle, hip, and elbow.

The second columns of the left and right poses show the estimations are improved. It is much closer to the third column of ground truth. So, fine-tuning can correct the errors from background interference, obscuring, and view inconsistency. In addition, it can be seen that the HR network’s estimation of hip and knee joints is compelled in a line, which is quite different from the HG’s estimation. That means the HR network can be further improved.

## 5. Conclusion

With the popularization of boxing in China, the lack of coaches and amusement impedes the promotion of this sport. The research on intelligent humanoid boxing robots becomes hotter, and the problem of insufficient coaches can be solved. Through the application of human pose estimation technology, the actions of boxing athletes can be analyzed, guided, and taught. The inconsistent inputs between the current image-based 2D human pose estimation technology and the 3D data of RGBD prevent our study because of the shortage of boxing data. The model transfer method is adopted to improve the technology application by patching the lack of channel. Three SOTA models of this technology were studied and transferred for experiments. Different strategies of transfer were examined to patch the lack of depth channel. The results show that the mean combination of RGB channel parameters is suitable to patch the depth channel. This strategy can improve models’ estimation performance stability. In addition, model transfer learning can efficiently reduce the dependence on collecting new data. The three SOTA models of the OP, HR, and HG networks exhibit competitive ability, and each model achieves a better performance after a mixture of depth channel information. Based on this research, the technical problems existing in the application of boxing can be revealed further, such as the HR network needing to improve the estimation of hip and knee joints and integrating these basic models into a small platform for the different kinds of applications. A machine learning method to optimize this combination can be researched further. Nonetheless, transfer learning with the channel patching method has been successfully studied for boxing pose estimation, and the 2D model performance can be improved by a 3D camera. Data can be collected to enhance the model’s application.

## Data availability statement

The original contributions presented in this study are included in the article/supplementary material, further inquiries can be directed to the corresponding author.

## Author contributions

JL supervised the projects, proposed the research plan for this article, and dealt with the revision of the algorithm. XC supervised the analysis of models and analyzed the theory for networks. XX and TH were in charge of data collection and analysis, coding, article writing, translation, and discussion. WW, SX, and CL implemented the experiments, analysis of results, and code revising. All authors contributed to the article and approved the submitted version.
